# Enriched Environment Exposure Enhances Social Interactions and Oxytocin Responsiveness in Male Long-Evans Rats

**DOI:** 10.3389/fnbeh.2018.00198

**Published:** 2018-09-05

**Authors:** Steven Neal, Molly Kent, Massimo Bardi, Kelly G. Lambert

**Affiliations:** ^1^Department of Psychology, Randolph-Macon College, Ashland, VA, United States; ^2^Department of Psychology, University of Richmond, Richmond, VA, United States

**Keywords:** enriched environment, natural habitat, use-dependent plasticity, oxytocin, corticosterone, acute stress, social interaction

## Abstract

Both social and physical stimuli contribute to the complexity of an animal’s environment, influencing biobehavioral responses to subsequent challenges. In the current study, male Long-Evans rats were randomly assigned to an isolate (ISO), social control (SC) or social enriched (SE) group (*n* = 8 per group). The SC and SE conditions were group housed with the SE group exposed to physical enrichment stimuli that were natural as opposed to manufactured (e.g., hollowed out log instead of plastic hiding place). On three occasions during their 40-day enriched environment exposure, night/dark phase videos were obtained for 1 h during the early part of the dark phase. During this time, the SE animals exhibited significantly more social grooming with no differences between the SE and SC in the frequency of play or self-grooming bouts. Subsequently, all animals were assessed in social interaction and problem-solving escape tasks during the last week of the enriched environment exposure. SE rats exhibited increased digging bouts toward the restrained conspecific in the social interaction task whereas the other groups exhibited more escape responses. In the problem-solving task, SE animals exhibited a decreased latency to cross the barrier to escape from the predator odor (i.e., cat urine and fur). Neural analyses indicated increased oxytocin-immunoreactive (OT-ir) tissue in the SE supraoptic and paraventricular nuclei of the hypothalamus compared to the other groups. Interestingly, blood samples indicated lower peripheral corticosterone (CORT) and higher OT levels in the ISO animals when compared to the SC and SE animals, an effect retrospectively attributed to separation anxiety in the SE and SC animals in preparation for histology procedures. When the behavioral, neural and endocrine data were visualized as a multifaceted dataset via a multidimensional scaling analysis, however, an association between social enrichment and higher OT involvement was observed in the SE animals, as well as heightened stress responsivity in the ISO and SC groups. In sum, the SE animals exhibited a facilitation of social responses, problem-solving ability and OT immunoreactive responsiveness. These findings provide new information about the influences of both physical and social stimuli in dynamic and enriched environments.

## Introduction

In the late 18th century, Italian anatomist, Michele Vincenzo Malacarne, reported that when animals underwent extensive training, they developed more cerebellar folds than untrained animals (described in Rosenzweig et al., 1972). These provocative findings were controversial and, consequently, laid dormant until the mid-20th century when a prominent Canadian neuroscience researcher, Donald Hebb, reported that rats raised in an engaging environment in his own home exhibited enhanced learning compared to his standard laboratory raised animals (Hebb, 1949). Subsequently, a team of neuroscientists at UC Berkeley demonstrated that, contrary to prevalent assumptions about the fixed nature of the nervous system, rodents exposed to a complex environment developed heavier cortical areas and altered neurochemistry (Rosenzweig et al., 1962; Bennett et al., 1964). Instead of spending an inordinate amount of time training the animals, it was assumed that the animals would engage in a form of self-training if they were housed in engaging environments (Rosenzweig and Bennett, 1996). These complex environments became known as “enriched environments” to contrast with the typical cages used in the laboratory (Krech et al., 1960; Diamond et al., 1965). Rather than being enriched, however, these complex environments may be closer to an ecologically relevant environment—that is, a standard environment by nature’s criteria (Juraska and Wise, 2015). Even so, the terms enriched and complex are used interchangeably throughout this manuscript to refer to engaging laboratory environments that include social and physical stimuli.

Early laboratory enriched environment studies utilized at least three groups: (1) an enriched social group with 8–10 animals housed in a large cage with objects that were rotated every few days; (2) a social control (SC) group of approximately three animals housed in a standard size cage; and (3) an isolate (ISO) control group with animals housed individually in a small cage (Rosenzweig et al., 1972). Compared to individual animals housed in standard laboratory cages, these enriched environments provided more diverse experiences for the animals with varied exposures to sensory, motor and social stimuli. Similar variations of these original groups are used in current studies; however, regardless of the size of the cage and social group, the physical stimuli are rotated every day or couple of days so that the animals experience novel conditions in their laboratory habitats (Nithianantharajah and Hannon, 2006).

The observed neurobiological effects of enriched environments have been both widespread and reliable. For example, the visual cortex increased approximately 6% in enriched animals (Bennett et al., 1964; Juraska et al., 1989) with more specific changes such as increased dendritic branching and spine density (Volkmar and Greenough, 1972; Globus et al., 1973). Although the volume changes are not as pronounced in the hippocampus (approximately 3%), increased dendritic spines have been observed in the absence of modifications in the dendritic tree in the dentate gyrus and CA3 area (Juraska et al., 1989; Moser et al., 1997). Enriched environments have also been shown to increase hippocampal neurogenesis rates (Kempermann et al., 1997), an effect that is enhanced when animals have the opportunity to engage in running behavior (Kobilo et al., 2011). Neurogenesis has been suggested as a mechanism for enhanced flexibility in the responses of animals housed in enriched environments, as varied responses are acquired in the dynamic, engaging environments (Garthe et al., 2016).

From the time that the earliest enriched environment studies were published, there has been an attempt to isolate the most influential underlying mechanisms of brain plasticity. Although adding environmental enrichment to single-housed animals has been shown to be beneficial to the animals’ wellbeing over long periods of time (Abou-Ismail and Mahboub, 2011), the extent of brain changes observed in social enriched (SE) animals (that is environmental enrichment and social housing) has not been replicated in the single-housed enriched animals (Rosenzweig et al., 1978). Additionally, it is generally thought that ISO-housed animals represent impoverished conditions that render the animals susceptible to the subsequent emergence of anxiety-related responses such as motor stereotypies (Balcombe, 2006). Even though the importance of social housing has been established for the well-being of animals, the neuroanatomical enrichment effects have been suggested to be dependent on the animals’ interactions with environmental stimuli (Rosenzweig et al., 1978). Considering that, since the original enriched environment studies were conducted, SC groups are often housed in the same size cage as the SE animals, the large cage size used in enriched environment studies has also been ruled out as the most important factor underlying the enriched environment effects (Lambert et al., 2016). Further, it has been acknowledged that many of the factors present in the enriched environment studies contribute to an *additive* effect on relevant neuroanatomical factors (Fabel et al., 2009).

Although the specific role of social responses in the enriched environment studies is still unknown, it has been suggested that playful social, physical and motor responses are critical for the neurobiological effects (Fagan, 1981, 1982). This play hypothesis was subsequently assessed when dark phase observations of the rats were evaluated to determine if the enriched, social animals engaged in more rough-and-tumble play than the standard SC animals. Interestingly, no differences in play behavior, or other measures of social interaction, were observed (Renner and Rosenzweig, 1986). Even so, the authors emphasized the importance of social housing since the social-housed control animals often exhibit brain effects that are intermediate between the SE environment and impoverished environment animals. A possible explanation for this putative additive effect may be related to local enhancement, or social facilitation, when an animal’s activity attracts the attention of conspecifics which may facilitate orientation and subsequent physical and social interactions with the observed environmental interaction (Thorpe, 1963; Renner and Rosenzweig, 1986). The neuropeptide oxytocin (OT), involved in social responses ranging from maternal care to social trust, is a plausible mechanism for social facilitation in SE environments (Veenema, 2012). Because OT has been associated with positive social interactions and reduced stress responsivity, it is a common target when investigating neurobiological mechanisms of affiliative social responses and accompanying enhancements in overall wellbeing (Uvnäs-Moberg, 1998).

In contrast to the previously mentioned study (Renner and Rosenzweig, 1986), when rats are exposed to an enriched environment with natural, as opposed to artificial, stimuli and observed during the dark phase, increased interactions with both physical and social stimuli have been observed. Further, compared to animals placed in an environment with artificial stimuli, the natural-enriched animals exhibited less anxiety-typical behavior in response to a predator odor (Lambert et al., 2016). In a similar study, natural enriched rats exhibited more evidence of emotional regulation in a challenging swim escape task, evidenced by shorter latencies and increased frequencies of diving responses as well as higher DHEA/corticosteroid ratios; however, no differences in hippocampal BDNF levels were observed between the natural- and artificial-enriched groups (Bardi et al., 2016). These findings confirmed earlier observations of the UC Berkeley team indicating more neuroanatomical modifications in enriched animals housed in a naturalistic outdoor habitat that provided opportunities for burrowing and exposed the animals to additional natural elements than encountered by the laboratory enriched animals (as described in Rosenzweig et al., 1972). If the natural environments stimulate more species-relevant responses (directed toward both physical and social stimuli), this habitat presents an optimal environment to investigate the influence of social interactions on neurobiological outcomes observed in various enriched environments (Thorpe, 1963; Renner and Rosenzweig, 1986; Bardi et al., 2016; Lambert et al., 2016).

Given the previous findings in our laboratory using natural-enriched environments, the purpose of the current study was to further explore the role of social interactions in laboratory enriched environments. Since increased social interactions have been observed in natural-enriched animals (group housed with natural physical enrichment), this type of enrichment was used in the current investigation. The natural-enriched group was compared to SC (group-housed with no physical enrichment) and ISO-housed (individual-housed with no physical enrichment) groups. Thus, the three groups were similar to the groups used in the classic enriched environment studies, with the addition of the natural elements in the enriched environment. The opportunity for social interactions was viewed as optimal in the natural-enriched animals, standard in the SC animals and absent in the ISO-control animals. Given the rich literature implicating OT in social behavior (Carter, 1998; Nelson and Panksepp, 1998), both central and peripheral OT activity were evaluated. Social responsiveness and problem-solving behaviors were also assessed, as well as peripheral corticosterone (CORT) levels. It was hypothesized that, as observed in past studies, the enriched animals would be most affected by their habitat exposure due to heightened social and physical interactions and would exhibit increased central and peripheral OT responsiveness, less stress responsiveness, heightened social attentiveness and more efficient problem-solving responses. It was anticipated that the focus on social interactions in the current study would provide meaningful information about the role of OT and social attentiveness in animals’ neurobiological responses to enriched environments.

## Materials and Methods

### Animals

**Figure 1:**
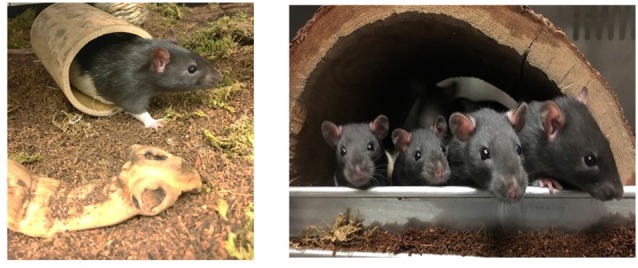
Images depicting the natural-enriched environments utilized in the current study.

Twenty-four male Long Evans rats were ordered from Envigo Laboratories (Indianapolis, Indiana) and arrived at 21–23 days of age. Rats were given 7 days to habituate to laboratory conditions before being assigned to one of three living environments: ISO, SC or SE groups. ISO rats were housed individually in cages (48 × 26 × 21 cm) with corncob bedding and food and water provided *ad libitum*. The SC group included eight males housed in a large cage (61 cm × 61 cm × 38 cm) containing a shallow floor pan for appropriate bedding substrate (corncob bedding), with food and water provided *ad libitum*. Provided as standard laboratory enrichment, 5 cm square nestlets (Ancare; Bellmore, NY, USA) were placed in the ISO and SC cages for the animals to manipulate. The SE group was housed in the same size cage as the SC animals, however, the bedding substrate consisted of shredded coconut husk substrate (Zoo Med Eco Earth; San Luis Obispo, CA, USA) that has the texture of dirt, as well as six patches of dried moss (Exo Terra; Mansfield, MA, USA). In addition to the natural bedding substrate, seven different objects (e.g., rocks, sticks, coconut shells) were placed throughout the SE cage (see Figure 1). The objects were either replaced or rotated within the cage every 4 days. Each object had an intended function (e.g., shelter, climbing, tunneling, or manipulating) which was maintained during changes. All three environments were kept on a 12-h light/dark schedule with lights on at 8 AM and lights off at 8 PM in a moderate temperature (approximately 22°C). This study was carried out in accordance with the recommendations of the Institutional Animal Care and Use Committee at Randolph-Macon College; further, the protocol was approved by the Institutional Animal Care and Use Committee at Randolph-Macon College.

### Behavioral Assessments

Behavioral observations of social interactions among the group-housed animals were videotaped three times throughout the duration of the study (at 1, 14 and 26 days after introduction to their respective environments). Each observation was videotaped for 1 h during the dark phase (8:30–9:30 PM). A red fluorescent light bulb was used to provide sufficient lighting to capture images of the animals during the dark phase while minimizing light-induced circadian disruptions.

Scan sampling was utilized to assess behavior during recordings. Briefly, every 30 s the social behavior of the animals was scored to determine the number of rats that were in contact with or within proximity of a conspecific. Each behavior was classified as active (physical activity) or passive (no observed movement). The number of animals in contact with each other, as well as, number of animals in close proximity (within one body-length) was also recorded. More detailed social behavior such as play and grooming were assessed by frequency and number of animals involved. A distinction was made in grooming behavior, noting bouts of self-grooming and social-grooming.

**Figure 2:**
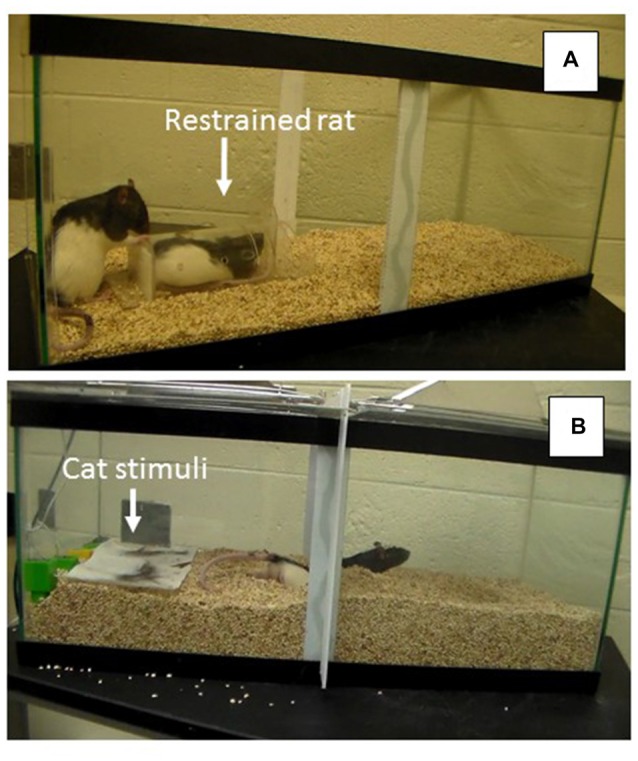
**(A)** Social Interaction Task depicting restrained rat in container and experimental rat exploring the container. **(B)** Problem-Solving Task depicting experimental rat escaping from the predator odor compartment by burrowing under the partition to reach the other side of the arena.

During the last week of assigned housing, animals were exposed to a social investigation task in which a novel male conspecific was placed in a plastic tube (22 cm × 0.9 cm × 6 cm), that allowed minimal movement of the animal. The tube had holes along the top to allow for the exchange of chemosensory (e.g., pheromonal) cues between the animal in the tube and the test animal placed in the aquarium (76.2 cm × 33 cm × 33 cm). The tube was placed on top of corncob bedding in one end of the aquarium with each of the 24 males placed at the opposite end of the tank during individual testing sessions (see Figure 2A). The stimulus males were rotated after every third session to avoid fatigue in the tube and to assure that each group was exposed to multiple stimulus males. The duration of the task was 7 min during which time a variety of behaviors were observed to assess social interest (i.e., latency to approach the tube, duration and frequency of tube investigation, frequency of tube manipulations (including digging around the tube, climbing on top of the tube and either touching and/or biting the tube), frequency of escape attempts, frequency of self-grooming bouts, frequency of sniffing bouts and duration of time spent in proximity (within 4 cm) of the tube).

A few days after the social investigation task, each of the 24 animals was also assessed in a problem-solving escape task involving the presence of multiple predator stimuli. For this assessment, rats were placed in an aquarium (76.2 cm × 33 cm × 33 cm) with corncob bedding and a partition in the middle of the tank that extended from the top of the tank to about 7 cm above the floor. During the habituation phase of training, animals had 5 min to explore the entire tank by easily walking under the partition to travel to the other side. During the test on the subsequent day, bedding was poured in the tank so that the space between the barrier and the floor was not apparent, requiring the rat to burrow through the bedding, under the partition, if they wanted to travel to the opposite side of the tank (see Figure 2B). During the problem-solving test, each animal was placed on one side of the partitioned aquarium that included cat urine and hair. Behaviors recorded during this 5-min test included: latency to burrow and dig under the partition and the frequency of digging bouts. All individual behavioral tasks were videotaped and coded so that each animal’s behavior could be subsequently analyzed by an observer blind to group assignment.

### Endocrine Assessments

To assess CORT and OT levels, blood samples were obtained prior to the perfusion of fixative solution through the cardiovascular system. Specifically, animals were anesthetized by exposure to 1 mL of Halothane liquid (Sigma-Aldrich; St. Louis, MO, USA) until respiratory rate slowed and were then given an intraperitoneal injection of 0.2 mL sodium pentobarbital at an overdose of 50 mg/Kg. Before the heart stopped beating, a needle was placed into the left ventricle and blood was slowly removed and stored in a −80°C freezer until the assays were conducted.

To assay for OT and CORT levels in blood samples, commercial kits were used (acquired from Enzo Life Sciences, Farmingdale, NY, USA). Before the assays, hormones were extracted by adding 1 mL of diethyl ether to each sample and then mixing the contents in glass tubes. Tubes were allowed to settle for about 30 min and then the aqueous phase was removed by freezing the samples at −80°C for a few minutes and then pouring the solution in a new tube. Subsequently, the ether was evaporated by placing the new tubes into a 42°C water bath inside a fume hood. The samples were reconstituted with the addition of assay buffer at the correct dilution for each hormone. The selected dilution was 1:20 for OT and 1:50 for CORT. Samples, controls and standards were prepared in duplicates and added to the appropriate wells of the assay kits, following the instructions provided in the kits. Sample readings were completed using an automated microplate reader (BioTek, Winooski, VT, model Synergy) and Gen5 software (BioTek, Winooski, VT, version 2.04.11). Readings were assessed at a wavelength of 405 λ with correction at 490 λ. Data were calculated using log-logit transformations of the absorbance values recorded from the reader, and analyzed by least-squared regression analysis. Accuracy was demonstrated at each standard curve point: all readings off the 5% threshold value from the standard curve were discarded. Quality control pools were assayed in triplicate on each plate. The sensitivity of the assays, as reported by the manufacturer, was 26.5 pg/mL for OT and 171 pg/mL for CORT.

### Histological Preparation

Following blood collection, animals were transcardially perfused at 40 mL/min using a MasterFlex L/S perfusion pump with phosphate buffered saline (PBS) followed by 4% paraformaldehyde. Following extraction, brains were post-fixed overnight with 4% paraformaldehyde at 4°C, then transferred to 10% sucrose solution for 24 h at 4°C followed by 20% sucrose at 4°C and finally into 30% sucrose at 4°C until time of sectioning. Brains were sectioned using a HM525 Microm cryostat in the anterior region (plate 46, Paxinos and Watcon, 2007) for assessment of the paraventricular and supraoptic nuclei (PVN and SON) of hypothalamus, as well as medial forebrain bundle (MFB) region. Six free-floating sections (40 μm) were collected, placed in PBS and prepared for immunohistochemistry.

For OT immunoreactivity assessment of the PVN, SON and the MFB, sections were washed in PBS to remove excess sucrose and paraformaldehyde. Sections were then incubated in 0.3% hydrogen peroxide for 10 min. Subsequently, the sections were blocked with 10% normal goat serum (Vector, Burlingame, CA, USA) in PBS-BT (BSA, Vector; Triton-X 100, Spectrum Chemical: Cardena, CA, USA) for 60 min before being incubated in the OT primary antibody (1:4,000 dilution, Immunostar, Inc., Hudson WI, USA) for 48 h at 4°C. Sections were subsequently washed in PBS-BT then exposed to the biotinylated secondary antibody for 90 min (goat anti-rabbit; 1:200 dilution, Vector). Following incubation, sections were processed with an Elite Vecastatin ABC kit (Vector). Finally, sections were visualized with DAB peroxidase substrate and then cleared through a series of 70, 95 and 100% ethanol followed by Citrasolv (Fisher Scientific, Fair Lawn, NJ, USA) and coverslipped with permount (Fisher Scientific).

### Neural Quantification

Prior to being analyzed all slides were recoded to ensure experimenters would be blind to experimental conditions. A BA400 light microscope (Miotic, Richmond, BC, Canada) was used for neuroquantification. To assess the area of OT immunoreactivity, cell bodies and fibers were thresholded using a 135 × 135 μm area at 40× magnification. The percent area stained was determined using light-thresholding software (Bioquant Life Sciences, Nashville, TN, USA).

### Statistical Analysis

To assess the combined effects of variables related to specific system outputs (oxytocin-immunoreactive (OT-ir) in different brain areas, and the peripheral levels of OT and CORT), MANOVA was used to test the overall effects of housing conditions (three levels: ISO, ES and CS groups). For the individual behavioral tasks, ANOVA was used to determine the effect of each of the three housing conditions on the behavioral output. The significance value for each analysis was set at *p* = 0.05. Following the analyses of variance, appropriate Tukey *post hoc* tests were conducted to identify the treatment group(s) responsible for the variation. During the dark phase observations in the control and enriched animals, the presence or absence of play behavior, self-grooming and social-grooming was recorded every 30 s for 1 h for three consecutive nights. Thus, a total of 360 data points were collected for each behavior. The marginal frequencies for binary output (presence/absence of focal behavior) was calculated for each of the three behavioral categories and compared among the three treatment groups. The likelihood ratio was used to determine if the frequencies in the two groups were significantly different.

To model the independent effects of housing conditions on each of the dependent variables, a non-parametric, ALSCAL, multi-dimensional scaling (MDS) model was used. It is important to remember that the main advantage of multivariate models is that the single contribution of each of the measures included in the analysis is independent of the other variable contributions. In other words, even if in itself a single measure appears to be higher in any given treatment condition, when the shared variance is partitioned out, the remaining individual contributions can provide a very different picture than provided by the initial mean values for a particular dependent measure. This is why multivariate models are essential in establishing associations in complex phenomena in which many different systems contribute to the final output. Generally speaking, MDS is a technique used to uncover the “hidden structure” to a set of data (Kruskal and Wish, 1978). To accomplish this, MDS generates graphical models that provide a spatial representation of the similarity structure of variables. Using the matrix of covariation among all the measures entered in the model, the relationships among variables can be displayed graphically. In order to map all of the variables into a desired space (two dimensional or greater), a certain lack of fit, referred to as the s-stress, is inevitable. The values of s-stress range from 0 (perfect fit) to 1 (worst possible fit). Thus, the aim of MDS is to find a map of the variables that minimizes the s-stress for a given number of dimensions. Kruskal’s s-stress values <0.15 are typically deemed acceptable, and below 0.1 indicates an excellent fit. Additionally, a good model needs to explain most of the variance present in the original data, which is expressed by the *R*^2^ value in the model. Typically, *R*^2^ values of 0.8 or higher are desirable, with values above 0.9 considered to be excellent.

## Results

### Neuroendocrine Results

**Figure 3:**
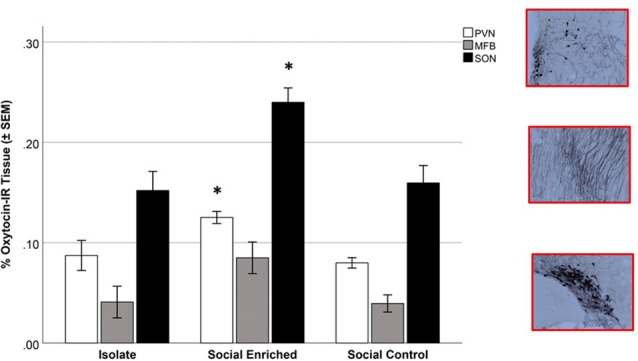
Oxytocin-immunoreactive (OT-ir) tissue in isolate (ISO), social enriched (SE) and social control (SC) animals in the paraventricular nucleus of the hypothalamus (PVN), supraoptic nucleus (SON), and medial forebrain bundle (MFB). In the PVN and SON areas, the SE animals had higher OT-ir measures than the ISO and SC groups (*p* < 0.05 for each area). No significant differences were observed in the MFB area. *Indicates significant difference from ISO and SC animals in comparable brain areas.

**Figure 4:**
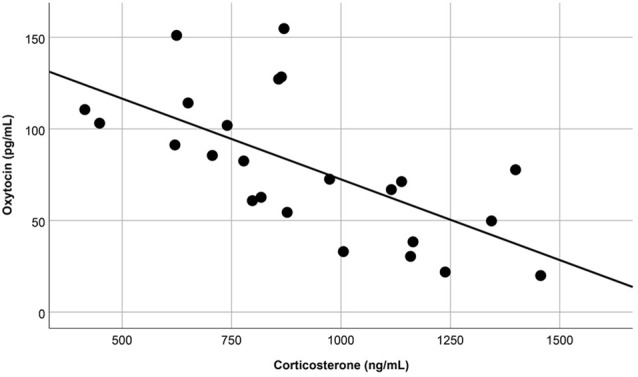
Blood samples revealed that, regardless of treatment group, peripheral OT and corticosterone (CORT) levels were inversely related (*p* = 0.01).

**Figure 5:**
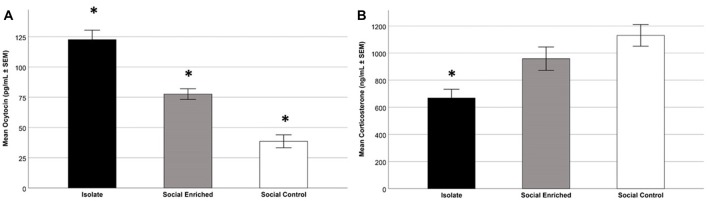
Mean plasma OT levels in the three treatment groups; specifically, the ISO animals had higher levels than the other groups and the SE animals had higher levels than the SC (SE) animals (*p* < 0.001; **A**). Focusing on mean plasma CORT levels in the three treatment groups; as depicted, the ISO animals had lower levels than the other groups (*p* < 0.05; **B**). *Indicates significant difference from other groups.

The overall OT-ir in all brain areas examined was significantly different among the three groups (Hotelling’s Trace = 0.972; *F*_(6,36)_ = 2.91; *p* = 0.020). Specifically, SE animals had higher OT-ir than both SC and ISO groups in the PVN and SON (Tukey *post hoc* test *p*-values < 0.032), whereas no significant difference was found in the MFB (all *p* = values > 0.073; Figure 3). Additionally, peripheral OT and CORT levels were inversely related (*r* = −0.65, *n* = 24, *p* = 0.01 (Figure 4). The overall peripheral OT activity was significantly different among the three groups (Hotelling’s Trace = 5.064; *F*_(4,38)_ = 24.05; *p* < 0.001). Specifically, ISO animals had the highest OT levels, whereas SC animals had the lowest (all Tukey* post hoc* test *p*-values < 0.001; Figure 5A). Conversely, ISO animals had significantly lower CORT levels than both SE and SC animals (all Tukey *post hoc* test *p*-values < 0.038). There was no significant difference between the SE and SC groups (Tukey *post hoc* test *p* = 0.279; Figure 5B).

### Behavioral Tasks

**Figure 6:**
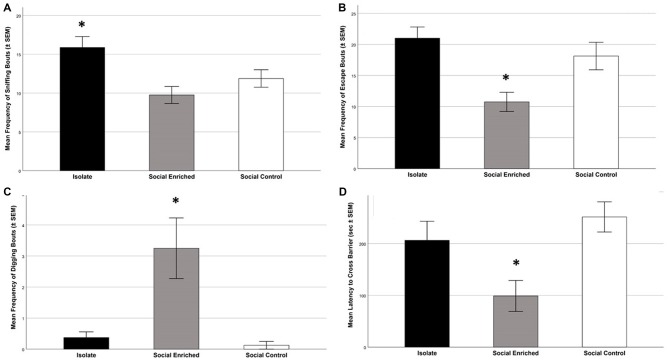
The behavioral tasks revealed higher sniffing bouts in the ISO animals than the other groups (**A**; *p* = 0.006); further the SE animals had lower escape frequencies than the other groups (*p* = 0.003; **B**) and higher frequencies of digging bouts (*p* = 0.001; **C**). Focusing on the problem-solving digging task, the SE animals had a lower latency to cross the barrier than the SC group (*p* = 008; **D**). *Indicates significant difference from other groups in **(A–C)**; *indicates significantly different from SC group in **(D)**.

During the Social Investigation Task, the latency to approach the tube and the frequency of total interactions with the tube (bite, paw, climb) were not significantly different by group (Latency: *F*_(2,21)_ = 2.36; *p* = 0.119; Interaction: *F*_(2,21)_ = 0.85; *p* = 0.441), but the frequency of sniffing the tube was higher in ISO animals (*F*_(2,21)_ = 5.52; *p* = 0.006; Figure 6A). Focusing on the additional behaviors, SE animals had the lowest number of escape attempts (*F*_(2,21)_ = 7.95; *p* = 0.003; Figure 6B) and the highest number of digging bouts directed toward the tube (*F*_(2,21)_ = 9.00; *p* = 0.001; Figure 6C).

During the Predator Stimuli Escape Task, the latency to escape from the side of the apparatus containing the predator stimuli to the other side was significantly different among the three groups (*F*_(2,21)_ = 5.95; *p* = 0.009; Figure 6D). Tukey *post hoc* tests indicated that the SE group was faster to escape than the SC group (*p* = 0.008), but there was no significant difference with ISO animals (*p* = 0.069). The number of digging bouts during the same task was not significantly different among the three groups (*F*_(2,21)_ = 1.91; *p* = 0.173).

### Dark Phase Observations

The marginal frequencies for binary output (presence/absence) of play behavior, self-grooming and social grooming were recorded during the dark phase observations (Table 1). No significant differences were found between the SE and SC groups for play behavior (Likelihood ratio = 0.139, *p* = 0.709) and for self-grooming (Likelihood ratio = 1.639, *p* = 0.201), but there was a significant difference observed in the social grooming data; specifically, individuals in the SE condition were approximately four times more likely to engage in social grooming than individuals in the SC housing condition (Likelihood ratio = 3.851, *p* = 0.048).

**Table 1:** Marginal frequency cross tabulations.

		Play SC		
Count		Absence	Presence	Total
Play SE	Absence	197	58	255
	Presence	83	22	105
Total		280	80	360
	**Value**	**df**	***p*-value**	
Likelihood Ratio	0.139	1	0.709	
		**SelfG SC**		
**Count**		**Absence**	**Presence**	**Total**
SelfG SE	Absence	71	94	165
	Presence	71	124	195
Total		142	218	360
	**Value**	**df**	***p*-value**	
Likelihood Ratio	1.639	1	0.201	
		**SG SC**		
**Count**		**Absence**	**Presence**	**Total**
SG SE	Absence	272	19	291
	Presence	64	5	69
Total		336	24	360
	**Value**	**df**	***p*-value**	
Likelihood Ratio	3.851	1	0.048	

*Numbers represent counts of behavior (absence/presence) in two groups: Social Control (SC) and Social Enriched (SE) during the night observation of spontaneous social behavior. Play, play behavior; SelfG, self-grooming; SG, social grooming*.

### Integrative Multivariate Model

**Figure 7:**
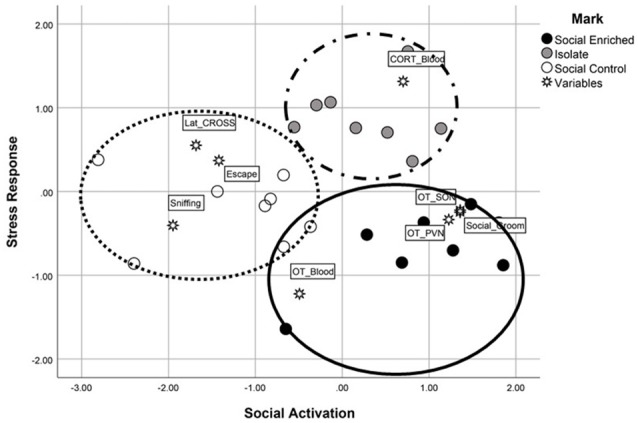
The multidimensional scaling analysis, an additional statistical evaluation analysis, revealed high discrimination among the three groups. Using the two linear dimensions of *social activation* and *stress responsiveness*, the SE animals were characterized by higher social activation and lower stress responsiveness (e.g., higher OT-immunoreactivity, social grooming) whereas the SC animals were characterized by lower social activation responses (e.g., higher number of escape attempts in the social investigation task) and higher stress responsivity (e.g., longer latency to cross the barrier in the problem-solving task). The ISO animals were limited in behavioral responses due to their lack of cagemates during the dark observation phase; however, when the shared variance was subtracted from the scores, their plasma CORT levels placed them high along the stress responsiveness dimension.

We mapped the multivariate, independent association among all the significant measures assessing different system outputs (neural, endocrine and behavioral) using a MDS model. The map, provided in Figure 7, clearly indicated that housing conditions modified the system outputs to such an extent that we were able to discriminate efficiently among the individual subjects. Discrimination rate reached a perfect 100%, since no individuals with different housing conditions were clustered together. Moreover, both measures of accuracy for the MDS model indicated an excellent fit (Kruskal’s stress index = 0.062; *R*^2^ = 0.97). The two dimensions created by the MDS model, both linear combinations of the dependent variables entered in the model, were named *Social Activation* (dimension 1) and *Stress Response* (dimension 2). It is important to point out that the ISO animals didn’t have the full scale of behaviors to contribute to the analysis due to their restricted housing environment (i.e., behaviors in their home cages during the dark phase were not recorded since they were ISO housed, a condition that was necessary for the experimental design of the current study). Animals exposed to the SE environment were characterized by a higher OT-ir activity in both the PVN and SON areas, as well as a higher probability to engage in social grooming during the dark-phase observations. Alternatively, SC animals were characterized by higher levels of sniffing and escaping during both the Social Investigation task and Predator Stimuli task. Finally, although it was unexpected considering the MANOVA results, ISO animals were characterized by higher levels of stress arousal—after removing the partial effects shared with the other measurements (see Figure 7).

## Discussion

In the current study, animals were housed in habitats designed to yield varying amounts of social engagement (i.e., isolate-housed (ISO; no social contact); social-control (SC; moderate social contact) and contact) and social-enriched (SE; high social contact)) to explore the effects of environmental variables on social- and predator-stress responsiveness. In general, the results corroborate and extend previous research investigating social behavior in enriched environments by exploring interactions between physical and social stimuli used in the enriched environment investigations, as well as potential neurobiological correlates. As observed previously in our laboratory, animals housed in enriched environments containing natural elements demonstrated seemingly adaptive responses in the predator escape task (Lambert et al., 2016). The social interaction task used in the current study revealed that SE animals demonstrated more digging directed toward the stimulus animal, as well as fewer attempts to escape, whereas the ISO animals engaged in more exploratory sniffing. Collectively, these responses indicated that the SE animals directed more attention toward the stimulus animal in the restraint tube whereas the ISO animals directed their attention away from the distressed animal. Increased social/affiliative contact in the form of social grooming, but not social play, was observed in the SE animals during the dark phase. Neurobiological data revealed increased OT immunoreactivity in the SE animals; however, contrary to the hypothesized results, plasma levels of OT were highest in the ISO animals during the last phase of varied habitat exposures. Further, plasma CORT was lowest in the ISO animals at this time, an effect attributed to the unexpected role of separation anxiety during the procedure of separating animals for the histological/perfusion process. Beyond the MANOVA and ANOVA results, the MDS analysis suggests that the SE environment was also associated with a less responsive HPA axis, or CORT levels when shared variance among the dependent variables was accounted for in the data set. The relevance of these findings, along with potential contributing factors, are discussed below.

Focusing on social behavior, the results are in alignment with the initial hypothesis that the SE environment would generate the highest levels of affiliative social behavior. During the dark phase observations of spontaneous behavior, several responses, including social play, self-grooming and social-grooming, were observed. Interestingly, the SE animals exhibited an approximately 350% increase in social grooming when compared to SC animals. In agreement with Renner and Rosenzweig (1986), the SE habitat did not affect bouts of rough-and-tumble play behavior. In contrast to the current results, Renner and Rosenzweig failed to find enriched-induced increases in grooming interactions. Based on previous findings in our laboratory, it is likely that the heightened social interactions in the current study were due to the natural elements in the enriched habitat (Lambert et al., 2015, 2016; Bardi et al., 2016). Even so, further research with both natural and artificial enriched environments will further elucidate the role of natural elements in social facilitation in enriched environments.

Behaviors observed in the social interaction task corroborated the dark phase observations. When a conspecific was placed in a Plexiglass tube, ISO rats exhibited behavior directed away from the conspecific (e.g., sniffing, trying to escape) whereas the SE animals exhibited more digging bouts directed toward the conspecific in the tube. Thus, the SE animals exhibited more interest in the animal than the habitat, evidence of increased social attentiveness. The observation that the SC animals scored in an intermediate range in the sniffing and escape responses suggests a potential additive effect of the enrichment to the traditional social housing, as previously described in animals exposed to both running exercise and enriched environments (Fabel et al., 2009). This heightened social interest may also be influenced by the previously described social facilitation effect proposed to explain the animals’ engagement in enriched environments; specifically, that animals direct their attention to other animals in the SE environment, resulting in increased attentiveness to the behavioral responses of conspecifics (Thorpe, 1963).

The neuropeptide OT has been implicated in the formation of social bonds, in addition to other important physiological functions such as lactation and parturition (Carter, 1998; Nelson and Panksepp, 1998). OT is produced in the supraoptic and paraventricular hypothalamic nuclei with pervasive projections throughout the brain, including limbic, diencephalon, mesencephalon, brainstem and spinal cord areas of the rodent brain (Sofroniew, 1983). The extent of OT binding in the limbic system of humans appears to be less extensive than rodent observations; additionally, increased binding has been observed in the basal forebrain and substantia nigra in the human brain (Stevens et al., 2013). Because the roles of central and peripheral OT are still being evaluated, it is important to consider both sources when possible (Gordon et al., 2010), as was done in the current study. Interestingly, very different results were observed with these two measures. The central measures indicated, as hypothesized, more OT-immunoreactivity in the PVN and SON in the SE groups than the other groups. However, the highest peripheral levels were observed in the ISO group. Although not anticipated as an influence, it is likely that this effect is due to the protocol used during the perfusion/blood collection process. During this procedure, animals were maintained in isolation for a few minutes prior to anesthesia. Although being in a novel environment prior to the procedure was recognized as a potential stressor for all animals; in retrospect, the two social-housed groups likely experienced unintended stress in the form of separation anxiety once they were removed from their cagemates. Thus, during this brief time prior to the onset of the anesthesia, the ISO animals may have experienced less stress than their social-housed counterparts. The peripheral CORT data suggest that the ISO animals were less stressed at that time (i.e., had lower CORT levels). This inverse relationship between peripheral OT and plasma cortisol levels has been previously observed in humans (Heinrichs et al., 2003). If the social housed animals experienced separation anxiety, then OT levels were likely affected since separation and attachment anxiety have been associated with low OT levels (Eapen et al., 2014). Focusing on CORT, however, past research suggests that, following early life isolation, single-housed male rats have lower CORT levels during recovery from stress than group-housed conspecifics (Lukkes et al., 2009). Further, dynamic social hierarchies in group housed rodents have been associated with higher CORT levels than observed in ISO-housed animals (Bronson, 1973). Although no aggressive social interactions were observed in group-housed animals in the current study, it is likely that individual differences existed due to the animals’ established social hierarchies (Beery and Kaufer, 2015). Regardless of the cause of the observed endocrine effects, these observations serve as a valuable reminder of the influence of social housing in laboratory animals at every stage of the experimental period.

Focusing on the central OT effects in the current study, the results indicated an interesting effect of enriched environments on central OT responsivity. A variation of this effect was observed in a previous study in which maternal rats housed in an enriched environment and exhibiting low-licking and grooming behavior resulted in their pups developing enhanced OT receptor binding than the pups raised by comparable mothers in a standard environment (Champagne and Meaney, 2007). Additionally, an effect of a limited enriched environment was observed in rats when exposure of single-housed animals to a nestlet (for nest building) accelerated wound healing in a pattern that was similar to the administration of OT (Vitalo et al., 2009). In the current study, the results are unique in that heightened measures of central OT and accompanying social behavior were observed in animals exposed to an enriched environment. Beyond the facilitation of social bonding, OT has also been implicated in the influence of experience-dependent, cross modal sensory experiences on the subsequent development of sensory cortical areas (Zheng et al., 2014). Accordingly, heightened engagement with both physical and social stimuli observed in the SE group may have been mediated through the oxytonergic system. Further, the increased OT-immunoreactivity observed in the SE animals may have had additional effects on the sensory cortical development of the animals. Expanding on the findings of increased OT immunoreactivity observed in the PVN of the hypothalamus in animals exposed to enriched environments, past research has confirmed that sensory deprivation leads to reduced OT-positive neurons in the same brain area (Zheng et al., 2014).

As the current study, and prior investigations in our lab, have confirmed, the addition of natural elements in the laboratory enriched environment habitats may provide valuable information about neural sensory-integration and healthy neural development (Bardi et al., 2016; Lambert et al., 2016). Because sensory integration therapies represent a common therapeutic approach for the treatment of neurodevelopment disorders (Green et al., 2006), a thorough analysis of cross-modality environmental interactions in preclinical models offers an opportunity to explore mechanisms in which complex environments facilitate the development of healthy brains. In contrast to enhanced sensory-integration, disruptions of sensory and motor development have been observed in several neurodevelopmental disorders such as Autism Spectrum Disorders (Reynolds et al., 2010). Thus, enriched environments, such as the natural enriched environments that heighten both physical and social interactions, provide a valuable opportunity to explore mechanisms leading to symptoms associated with neurodevelopmental disorders. Further, the timing and associated expectations of environmental changes may be relevant; for example, rats in a valproic acid model of enhanced vulnerability of autism disorders were less likely to develop hyper-emotionality symptoms if they were exposed to a predictable enriched environment as opposed to an unpredictable enriched environment (Favre et al., 2015). Because the focus of the current study was the opportunity for social interactions, the natural-enriched environment was utilized based on previously described results indicating heightened social interactions in our laboratory (Bardi et al., 2016; Lambert et al., 2016); however, the continued use of comparable artificial environments are necessary to determine specific differences between the natural and artificial elements of the environments.

The rich accumulation of data accrued from the vast array of research investigating various aspects of enriched environments has persistently demonstrated neurobehavioral effects, especially related to cognitive effects and cortical neuroplasticity (Juraska et al., 1989). In the current study, the enriched environment, perhaps due to the utilization of natural stimuli, provided an environment that produced enhanced OT-related functions in comparison to the laboratory standard and control group-housed animals. In addition to enhancement of neural processes, research has also consistently indicated that enriched environments offer protection against the onset of disorders of the nervous system such as Alzheimer’s Disease, Parkinson’s Disease, amyotrophic lateral sclerosis (ALS), as well as enhanced recovery from brain trauma (Nithianantharajah and Hannon, 2006). Further, enriched environments have also ameliorated symptoms of psychiatric illnesses such as depression and addiction in rodent models (Puhl et al., 2012; Richter et al., 2013; Grippo et al., 2014). Increased social interactions observed in the SE group during the spontaneous dark phase observations and the social investigation task were associated with increased OT-positive cells in the hypothalamus, OT cells that may play a role in cross modal sensory integration important for healthy brain functions. One limitation of the current study, however, is the use of only male animals. Because sex-dependent effects of the enriched environments have been previously observed, future research should consider both males and females to obtain a more comprehensive evaluation of the effects of social enhanced environments (Kolb et al., 2003; Bakos et al., 2009). Looking to the future, urther research is necessary to characterize maximal engagement with the environment to more fully understand the impact of complex environments on adaptive neural functions as well as enhance the translational value of these studies.

## Author Contributions

KL designed the study, supervised data collection and prepared the manuscript. SN contributed to the study design, collected data and contributed to writing the manuscript. MK supervised data collection, managed the data files and contributed to writing the manuscript. MB contributed to endocrine data collection, statistical analysis and writing of the manuscript.

## Conflict of Interest Statement

The authors declare that the research was conducted in the absence of any commercial or financial relationships that could be construed as a potential conflict of interest.
